# Risk factors and management of pulmonary infection in elderly patients with heart failure: A retrospective analysis

**DOI:** 10.1097/MD.0000000000027238

**Published:** 2021-09-24

**Authors:** Qi Peng, Qin Yang

**Affiliations:** a Cardiac Surgery, Wuhan Asia Heart Hospital, Jianghan District, Wuhan, Hubei, China; b Pharmacy Intravenous Admixture Services, The Sixth Hospital of Wuhan, Affiliated Hospital of Jianghan University, Jiangan District, Wuhan, Hubei, China.

**Keywords:** bacteria, heart failure, nursing care, prevention, pulmonary infection, treatment

## Abstract

Pulmonary infection is common in patients with heart failure, yet the risk factors remain unclear. We aimed to evaluate the clinical characteristics and risk factors of pulmonary infection in elderly patients with heart failure, to provide reference to the prevention of pulmonary infection.

This study was a retrospective study design. We included elderly heart failure patient admitted to our hospital from April 1, 2018 to August 31, 2020. The characteristics and clinical data of pulmonary infection and no infection patients were assessed. Logistic regression analyses were conducted to identify the risk factors of pulmonary infections in patients with heart failure.

A total of 201 patients were included. The incidence of pulmonary infection in patients with heart failure was 23.88%. There were significant differences in the age, diabetes, New York Heart Association (NYHA) grade, left ventricular ejection fraction (LVEF), C-reactive protein (CRP) between infection and no infection group (all *P* < .05), and there were not differences in the sex, body mass index, alcohol drinking, smoking, hypertension, hyperlipidemia, length of hospital stay between 2 groups (all *P* > .05). Logistic regression analyses indicated that age ≥70 years, diabetes, NYHA grade III, LVEF ≤55%, and CRP ≥10 mg/L were the independent risk factors of pulmonary infections in patients with heart failure (all *P* < .05). *Pseudomonas aeruginosa* (34.48%), *Staphylococcus aureus* (19.57%), and *Klebsiella pneumoniae* (15.22%) were the most common 3 pathogens in patients with pulmonary infection.

Heart failure patients with age ≥70 years, diabetes, NYHA grade III, LVEF ≤55%, and CRP ≥10 mg/L have higher risks of pulmonary infections, preventive measures targeted on those risk factors are needed to reduce pulmonary infections.

## Abstract 2

Supplemental Digital Content is available in the text

## 1 Introduction

Heart failure is mainly a clinical syndrome caused by ventricular insufficiency, which is associated with decreased cardiac output.^[1]^ Studies^[2,3]^ have shown that infection is the most important cause of heart failure. Elderly people are very prone to infection due to their weakened body immunity.^[4]^ Pulmonary infection is the main cause of death from heart failure in the elderly.^[5]^ It has been reported that heart failure patients with pulmonary infection have 2.5 times risk of death of those patients without pulmonary infection.^[6]^ Therefore, early diagnosis and prevention of pulmonary infection in elderly patients with heart failure is the key to treatment.

Heart failure is a common clinical manifestation of most organic heart diseases that progress to the end stage.^[7]^ It is mostly caused by the loss of ventricular pumping ability due to changes in cardiac structure and function.^[8]^ In contemporary society with an increasingly aging population, heart failure occurs greatly.^[9]^ The rate of heart failure shows an upward trend year by year.^[10]^ Patients with heart failure are often accompanied by secondary conditions such as pulmonary circulatory congestion and pulmonary edema, which can lead to dyspnea, gas exchange disorders, and other consequences, creating certain conditions for pathogens to invade and colonize the lungs.^[11,12]^ Therefore, patients with heart failure may have higher risk of pulmonary infection. Besides, pulmonary infection and insufficient oxygen uptake can increase pulmonary artery pressure, decrease the body's metabolic function, and further increase the burden on the heart.^[13]^ Presently, the potential influencing factors of pulmonary infection in patients with heart failure remain unclear. Therefore, we aimed to identify the risk factors of pulmonary infection in elderly patients with heart failure, to provide evidences to the treatment and nursing care of heart failure, thereby improving the prognosis of patients with heart failure.

## 2 Methods

We aimed to report this study in comply with Strengthening the Reporting of Observational studies in Epidemiology statement. Our study had been checked and approved by the ethical commissions of our hospital (SZC0018093), and all the included patients had signed the written informed consents.

### 2.1 Patients

We retrospectively analyzed the relevant clinical data of elderly heart failure patient admitted to the department of cardiac surgery, Wuhan Asia Heart Hospital (Wuhan, China) from April 1, 2018 to August 31, 2020. The inclusion criteria were: ① The clinical manifestations and echocardiographic examination results met the relevant diagnostic criteria for heart failure;^[14]^ ② The patients were ≥60 years’ old; ③ All patients received standardized anti-heart failure and anti-infective treatment of Ceftriaxone 2 g/day for 2 days; ④ All patients were informed and willing to participate in this study. The exclusion criteria of this study are: ① Patients with cardiogenic shock, acute and chronic organophosphate poisoning, pneumonia lung tumors, and autoimmune diseases at the timing of hospital admission; ② Patients with chronic lung diseases, lung malignant tumors, severe metabolic acidosis with a pH of 7.20 to 7.25 and bicarbonate 7 to 14 mmol/L, liver dysfunction (Child-Pugh level C) and kidney dysfunction (Creatinine >177 μmol/L), and patients who had used immunosuppressive drugs within 3 months before enrollment. ③ The patients who did not agree to take part in this study.

### 2.2 Diagnostic criteria for pulmonary infection

Patients need to meet ≥2 of the following criteria to be diagnosed with pulmonary infection:^[15]^① body temperature>38°C, duration of 4 to 6 days; ② white blood cell count >10 × 10^9^/L; ③ 3 consecutive sputum examinations showed the presence of pathogenic bacteria, and increased thick sputum; ④ computed tomography examination of the lungs showed the presence of inflammatory lesions.

### 2.3 Data collection

Our study was a retrospective study design, we retrospectively collected the data from the medical records and we all conducted follow-up until the discharge of patients. We calculated on the sample size following formula for the rate comparison of two groups^[16]^ based on the incidence of pulmonary infection reported in previous study,^[17]^ a sample size of 186 would be enough for the detection of group differences. We collected the following personal characteristics and clinical data of included patients, including sex, age, body mass index, alcohol drinking, smoking, hypertension, diabetes, hyperlipidemia, New York Heart Association (NYHA) grade, intubation, death in hospital, and length of hospital stay. The detection method of C-reactive protein (CRP) was as following: we took fasting venous blood from the patient in the morning, separated the serum, and detected by immunoturbidimetry. Left ventricular ejection fractions (LVEFs) were measured with a Philips heart color ultrasound detector (Philip ×300, German) after the patient was admitted to the hospital. Two authors used a unified form to gather patient-related information, which they then checked against each other to make sure it was accurate, to reduce the risk of biases.

### 2.4 Pathogen detection of pulmonary infection

We collected respiratory secretions specimens of patients in the infection group and used the MINGXI automatic microbial identification system (XD200, Shanghai, China) to detect the distribution of pathogens.

### 2.5 Data analysis

In this study, SPSS 19.0 software was used for related data processing (SPSS Inc, Chicago, IL). We have checked the normality of the analyzed data before choosing parametric analysis. Measurement data were expressed as mean ± standard deviation; comparisons between groups were examined by t test. Count data were expressed by percentage, and comparison between groups was examined by *χ*^2^ test. Logistic regression analyses were conducted to identify the risk factors of pulmonary infections in patients with heart failure. In this study, *P* < .05 was regarded as the group difference had statistical significance.

## 3 Results

### 3.1 The characteristics of included patients

A total of 201 patients with heart failure were included, of whom 48 had been diagnosed with pulmonary infection; the incidence of pulmonary infection in patients with heart failure was 23.88%. As indicated in Table 1, there were significant differences in the age, diabetes, NYHA grade, LVEF, and CRP between infection and no infection group (all *P* < .05), and there were no differences in the gender, body mass index, alcohol drinking, smoking, hypertension, hyperlipidemia, intubation, death in hospital, and length of hospital stay between 2 groups (all *P* > .05).

**Table 1:** The characteristics of included patients.

Variables	Infection group (n = 48)	No-infection group (n = 153)	*t*/*χ*^2^	*P*
Male	30 (62.50%)	98 (64.05%)	1.052	.081
Age, y	75.84 ± 8.11	68. 95 ± 8.46	1.746	.027
BMI, kg/m^2^	23.33 ± 1.03	22.9 ± 1.83	1.024	.106
Alcohol drinking	31 (64.58%)	90 (58.82%)	1.242	.085
Smoking	22 (45.83%)	77 (50.33%)	1.337	.094
Hypertension	27 (56.25%)	82 (53.59%)	1.601	.067
Diabetes	32 (66.67%)	40 (26.14%)	1.841	.009
Hyperlipidemia	17 (35.42%)	51 (33.33%)	1.197	.094
NYHA grade				
II	6 (12.50%)	38 (24.84%)		
III	12 (25%)	104 (67.97%)	1.182	.003
IV	30 (62.50%)	11 (7.19%)		
LVEF (%)	49.05 ± 12.34	61.28 ± 10.83	6.204	.014
CRP, mg/L	18.54 ± 5.49	6.44 ± 2.06	1.028	.009
Intubation	4 (8.33%)	10 (6.54%)	1.211	.052
Death in hospital	1 (2.08%)	3 (1.96%)	1.059	.088
Length of hospital stay, days	10.13 ± 3.51	9.58 ± 3.97	1.112	.075

BMI = body mass index, CRP = C-reactive protein, LVEF = left ventricular ejection fraction.

### 3.2 The risk factors of pulmonary infections in patients with heart failure

The variable assignment of multivariate logistic regression was presented in supplementary file 1, http://links.lww.com/MD/G407. As presented in Table 2, logistic regression analyses indicated that age ≥70 years, diabetes, NYHA grade III, LVEF ≤55%, and CRP,≥ 10 mg/L were the independent risk factors of pulmonary infections in patients with heart failure (all *P* < .05).

**Table 2:** Logistic regression analysis on the risk factors of pulmonary infections in patients with heart failure.

Variables	β	SE	OR	95% CI	*P*
Age ≥70 y	0.135	0.129	2.049	1.121∼3.208	.042
Diabetes	0.132	0.115	2.129	1.093∼3.042	.025
NYHA grade ≥III	0.108	0.099	2.455	1.264∼4.085	.017
LVEF≤55%	0.172	0.173	3.806	1.184∼6.102	.023
CRP ≥10 mg/L	0.141	0.102	1.934	1.016∼2.774	.041

CI = confidence interval, CRP = C-reactive protein, LVEF = left ventricular ejection fractions, OR = odds ratio, SE = standard error.

### 3.3 Pathogen distributions in elderly patients with pulmonary infection

Of the 48 patients with pulmonary infection, we have detected cases of pathogens; we could not identify the pathogens of the other two cases. As showed in Table 3, *Pseudomonas aeruginosa* (34.48%), *Staphylococcus aureus* (19.57%), and *Klebsiella pneumoniae* (15.22%) were the most common 3 pathogens in patients with pulmonary infection.

**Table 3:** The pathogen distributions in elderly patients with heart failure.

Pathogens	Cases	Percent
Gram-positive bacteria	14	30.44%
*Staphylococcus aureus*	9	19.57%
*Staphylococcus haemolyticus*	3	6.52%
*Staphylococcus epidermidis*	2	4.35%
Gram-negative bacteria	29	63.04%
*Pseudomonas aeruginosa*	10	34.48%
*Klebsiella pneumoniae*	7	15.22%
*Acinetobacter baumannii*	6	13.04%
*Enterobacter cloacae*	4	8.69%
*Enterobacter aerogenes*	2	4.35%
Fungus	3	6.52%
*Candida albicans*	3	6.52%

## 4 Discussions

With the increase of cardiovascular diseases and the aging of the population, the incidence of heart failure has gradually increased, of which 62.18% are elderly patients with heart failure.^[18]^ Pulmonary infection can increase pulmonary circulatory resistance, increase the afterload of ventricular contraction, and aggravate heart failure. It is the main cause of death in elderly patients with heart failure.^[19]^ Therefore, it is very important to judge whether elderly patients with heart failure are complicated by pulmonary infection.^[20]^ At present, the diagnosis of heart failure combined with lung infection is mainly through imaging examination and bacterial culture.^[21]^ Compared with imaging studies, bacterial culture can confirm whether there is pulmonary infection.^[22]^ The results of our study have showed that the incidence of pulmonary infection in patients with heart failure is 23.88%, and age ≥70 years, diabetes, NYHA grade III, LVEF ≤55%, and CRP ≥10 mg/L are the independent risk factors of pulmonary infections in patients with heart failure, which may provide reliable evidences to the management of heart failure.

In elderly patients with heart failure, the left ventricular myocardial contractility is weakened and the ejection ability is reduced, which often leads to pulmonary hypertension, pulmonary edema, and pulmonary congestion.^[23]^ Besides, in elderly patients, various functions of the body are reduced, bronchial glands and mucosal atrophy, and airway barrier function is weakened.^[24,25]^ Decreased alveolar elasticity and decreased mobility of the mucocilia in the trachea, all of which lead to a decline in immune function and a decrease in cough reflex sensitivity in the elderly, which makes the elderly heart failure more likely to merge with lung infection.^[26,27]^ Inflammatory factors stimulate pulmonary small blood vessels during lung infection, causing vasculitis, resulting in thickening of the vessel wall and narrowing of the official cavity.^[28]^ Meanwhile, inflammatory exudation increases the pressure in the alveoli, of which lead to varying degrees of pulmonary hypertension and increase the afterload of ventricular contraction.^[29]^ However, elderly patients often lack specific symptoms and signs due to the decline of the body's stress ability, which brings difficulties to clinical diagnosis.^[30]^ Meanwhile, patients with heart failure often have different degrees of pulmonary edema.^[31]^ It is difficult to diagnose the lungs through clinical lung auscultation.^[32]^ Therefore, it is difficult to determine whether it is complicated with lung infection. In the process of clinical treatment, doctors also rely on clinical experience to use antibiotics, which is prone to bacterial resistance.^[33]^ Therefore, early identification of risk factors for pulmonary infection is of great significance.

Elderly heart failure combined with pulmonary infection is a common condition in clinical settings.^[34]^ The severity and prognosis of heart failure and lung infection are also closely related.^[35]^ For patients with pulmonary infection, clinicians generally adopt empirical antibiotic treatment, but it is often accompanied by the overuse of antibiotics and the production of drug-resistant bacteria, and it brings unnecessary economic burden to patients, so understand that the distribution of pathogenic bacteria in patients with pulmonary infection is of great significance for guiding the choice of clinical treatment strategies.^[36]^ The pulmonary infections of patients with heart failure are mainly *Staphylococcus aureus*, *Klebsiella pneumoniae*, and *Pseudomonas aeruginosa*, which are consistent with the results of previous related studies,^[37,38]^ suggesting that the sputum of patients with heart failure and lung infection needs to be cultured. Therefore, the use of antibiotics in the treatment of elderly patients with heart failure and pulmonary infection should mainly inhibit Gram-negative bacteria. And we should use medicines reasonably according to the specific pathogenic bacteria infection to effectively control pulmonary infection.

Elderly patients with heart failure complicated with lung infection should take relevant preventive measures. Strictly monitor the sputum and bacteria culture of patients during treatment, and instruct them to eat more foods with high calories, high protein, and high vitamins, instruct patients who stay in bed for rest to properly raise the head of the bed and encourage their family members to help them get out of bed.^[39,40]^ Besides, back pat, expectoration, turn over times is beneficial to the prevention of pulmonary infection.^[41]^ And patients should be carried out oral care regularly, rinse mouth with 0.9% sodium chloride or 0.3% soda after meals to inhibit the growth of fungi.^[42]^ Patients with a history of smoking are advised to quit smoking, and patients with diabetes should strengthen blood sugar management and correct their wrong lifestyle.^[43,44]^ Those measures may be effective to the prevention of pulmonary infection in patients with heart failure.

Several limitations in this study must be considered. First, the sample size is small, it may lack sufficient power to detect the potential group differences, it will need more such studies with more participants to validate the findings. Secondly, our study is a retrospective design, we have failed to show the severity of pulmonary infection limited by collected data, many other factors that may influence the occurrence of pulmonary infection cannot be included for analysis. Therefore, we think that it may be underpowered to build a model to predict infection in heart failure population. Besides, together with the prediction model, a nomogram will be useful for the ease of clinical utility. Future studies with prospective design and more potentially associated variables in different areas and populations are needed. Thirdly, we have ruled out COVID-19 patients; during the COVID-19 pandemic, all the patients with positive COVID-19 would send to designated hospital for treatment in Wuhan, China. It has been reported that COVID-19 infection is associated with higher risks of pulmonary infection and poor prognosis.^[45–47]^ Therefore, more studies on the heart failure patients with COVID-19 infection are warranted.

## 5 Conclusions

To sum up, the pulmonary infection in patients with heart failure is very common, and the main pathogenic bacteria in elderly patients with pulmonary infection are Gram-negative bacteria, anti-Gram-negative bacteria should be used for antibiotic treatment when the pathogen infection is unknown. Besides, we have found that age ≥70 years, diabetes, NYHA grade III, LVEF ≤55%, and CRP ≥10 mg/L are the independent risk factors of pulmonary infections in patients with heart failure. Clinically, early prevention and intervention measures should be taken for these risk factors to reduce pulmonary infections.

## Author contributions

Q Y designed research; Q P, Q Y conducted research; Q P analyzed data; Q P and Q Y wrote the first draft of manuscript; Q Y had primary responsibility for final content. All authors read and approved the final manuscript.

**Conceptualization:** Qin Yang.

**Data curation:** Qi Peng, Qin Yang.

**Formal analysis:** Qi Peng, Qin Yang.

**Investigation:** Qi Peng, Qin Yang.

**Methodology:** Qi Peng.

**Project administration:** Qi Peng, Qin Yang.

**Resources:** Qin Yang.

**Supervision:** Qin Yang.

**Visualization:** Qin Yang.

**Writing – original draft:** Qin Yang.

## Supplementary Material

SUPPLEMENTARY MATERIAL
