# Supplementary material for: Risk factors and management of pulmonary infection in elderly patients with heart failure: A retrospective analysis
Source: Medicine (Baltimore). 2021 Sep 24;100(38):e27238. doi: 10.1097/MD.0000000000027238 (PMC10545257; doi:10.1097/MD.0000000000027238)
Supplement: SUPPLEMENTARY MATERIAL [file medi-100-e27238-s001.docx]

Supplementary file 1 The variable assignment of multivariate logistic regression

| Factors | Variables | Assignment |
| --- | --- | --- |
| Infection | Y | yes=1, no=2 |
| Age(y) | X_1_ | <70=1, ≥70=2, |
| Diabetes | X_2_ | yes=1, no=2 |
| NYHA grade | X_3_ | ⅠⅠ=1, III =2, Ⅳ=3 |
| LVEF | X_4_ | ≥55%=1, <55%=2 |
| CRP | X_5_ | <10 =1, ≥10=2 |
